# Clinical characteristics of dysplasia in UC and the correlation between dysplasia and UC recurrence

**DOI:** 10.48101/ujms.v131.13676

**Published:** 2026-02-02

**Authors:** Rongli Liu, Jing Luan, Qianbo Dong, Mingwei You, Yuanming Kang, Wenyan Wang, Hongli Liu, Liqun Yan

**Affiliations:** Department of Medical Imaging, Luquan Branch, The Second Hospital of Hebei Medical University, Shijiazhuang, China

**Keywords:** Ulcerative colitis, dysplasia, influencing factors, recurrence, correlation

## Abstract

**Objective:**

This research aims to identify the main influencing factors for dysplasia in ulcerative colitis (UC) patients and explored the correlation between dysplasia and the recurrence frequency of UC.

**Methods:**

This study retrospectively included 348 UC patients from the outpatient clinic of the Second Hospital of Hebei Medical University between December 2021 and December 2023 as the research subjects. The UC patients were divided into the dysplasia group and the non-dysplasia group based on pathological results. The general data and clinical data of the two groups were compared, and the typical computed tomography enterography (CTE) imaging features of the patients in the dysplasia group were explored. The main influencing factors for the occurrence of dysplasia in patients were screened using the univariate logistic regression analysis method and the ridge regression analysis method. All the follow-up data of the research subjects were complete, the recurrence situations of UC in the two groups were compared. The correlation between dysplasia and the recurrence frequency of UC was analysed using the Spearman rank correlation coefficient.

**Results:**

This study found that there were statistically significant differences in indicators such as disease classification, disease severity, lesion extent, disease activity degree, and faecal calprotectin (FC) level between the two groups of patients. Patients with dysplasia presented usually with CTE imaging features of mesenteric lymphadenopathy, mucosal abnormal enhancement, and bowel wall thickening. Univariate logistic regression revealed that the above indicators were influencing factors for dysplasia in UC patients (*P* < 0.05). Collinearity test and ridge regression analysis showed that chronic continuous type/acute fulminant type disease classification, severe disease severity, E3 lesion range, moderately active stage/severely active stage of disease activity degree, and high FC level would increase the possibility of dysplasia (*P* < 0.05). The recurrence rate of UC diseases in the dysplasia group was higher than that in the non-dysplasia group, and recurrence types were also different (*P* < 0.05). Spearman rank correlation analysis indicated that the grade of dysplasia, disease severity, lesion range, and degree of disease activity were positively correlated with recurrence frequency of UC (*P* < 0.05).

**Conclusion:**

Disease classification, disease severity, lesion extent, disease activity degree, and FC were independently correlated with occurrence of dysplasia in UC patients. Moreover, dysplasia increased the probability of patient recurrence. The grade of dysplasia and related influencing factors showed a positive correlation with the recurrence frequency of UC.

## Introduction

Ulcerative colitis (UC), a form of inflammatory bowel disease (IBD), is characterised by chronic and recurrent inflammation of the colon. With the changes in lifestyle and environment, the disease burden of UC is undergoing significant alterations worldwide (1). In Brazil, the prevalence of UC rose from 17.31 per 100,000 in 2012 to 84.23 per 100,000 in 2022 (2). Between 2010 and 2019, the prevalence in the United States (US) rose from 158 per 100,000 to 233 per 100,000 (3). The prevalence of UC in developed countries is several times to tens of times higher than that in developing countries, mainly due to economic development levels, lifestyle, and genetic factors (4). The main clinical manifestations of UC include diarrhoea, mucus and bloody stools, and abdominal pain (5). As the disease progresses, the intestinal mucosa of UC patients is stimulated by long-term chronic inflammation. The epithelial cells undergo repeated damage and repair, resulting in abnormal differentiation of intestinal mucosal cells in all UC patients to varying degrees. There is a probability of approximately one-tenth of developing into dysplasia (6, 7).

Dysplasia is a key stage of precancerous lesions and an important predictive indicator for the occurrence of colitis-associated colorectal cancer (CAC) in UC patients (8). Upon histological examination, dysplasia is categorised as indefinite (IND), low-grade (LGD), or high-grade (HGD) (9). Its occurrence is related to factors such as the disease course, the extent of the lesion, the degree of inflammation, and genetic susceptibility (10). However, a systematic understanding of the factors influencing dysplasia development in specific UC patients remains limited.

Currently, the relationship between dysplasia and recurrence is an important topic in clinical research. Even if UC patients receive appropriate treatment when they develop dysplasia, there is still a possibility that the disease may recur (11). There exists a complex interaction mechanism between dysplasia and recurrence, which may involve multiple factors such as persistent inflammation, abnormal molecular pathways, and therapeutic interventions (12, 13). The relationship between dysplasia and recurrence types still requires further clarification.

Therefore, this study aimed to investigate the influencing factors of dysplasia in UC patients and its impact on disease recurrence, and further evaluate the correlation between the grading of dysplasia and related influencing factors and the types of disease recurrence. This is expected to offer a guide for the optimisation of monitoring strategies and treatment for UC patients.

## Materials and methods

### Selection of patients

This study retrospectively enrolled 348 UC patients from the outpatient clinic of our hospital from December 2021 to December 2023 as the study participants. According to whether the UC patients had dysplasia in the pathological result, the UC patients were split into dysplasia group and non-dysplasia group. All the UC patients included in the study were followed up for 1 year, and the recurrence situation was statistically analysed. The recurrence situation was classified into three types: sporadic type (frequency of recurrence ≤1 time per year), frequent type (frequency of recurrence ≥ 2 times per year), and chronic continuous type (with continuous activity of UC symptoms and no relief).

The conduct of this study complies with the requirements of the World Medical Association’s ‘Helsinki Declaration’. It has been approved by the Ethics Review Committee of our hospital (Approval Number: 2025-R645).

### Calculation of sample size

The sample size was estimated using PASS 15.0 (PASS 15.0 Power Analysis and Sample Size Software. 2017. NCSS, LLC. Kaysville, Utah, USA. ncss.com/software/pass). The detection rate of dysplasia in UC patients was 15.8%, and the expected recurrence rate (RR) was 1.76. Assuming a two-sided test with α = 0.05 and δ = 0.2, the required total sample size was at least 348 cases, with at least 58 cases in the dysplasia group and at least 290 cases in the non-dysplasia group.

### Diagnostic criteria of diseases

Diagnostic criteria for UC: The patient was diagnosed with UC in the *Chinese Clinical Practice Guideline on Management of Ulcerative Colitis (2023, Xi’an)* (14): 1) Symptoms that have the typical clinical manifestations of UC require further examination to make a definite diagnosis; 2) Those who possess typical characteristics of colonoscopy can be clinically diagnosed as such; 3) If, in addition, the pathological examination of the biopsy specimen or the surgical resection specimen indicates the characteristic changes of UC, a diagnosis can be made; 4) For initial cases, if the clinical manifestations, colonoscopy findings and histological changes of the biopsy are not typical, UC should not be diagnosed immediately. Instead, a close follow-up should be conducted, and endoscopic and pathological histological re-examinations should be performed after a certain period of time (3 to 6 months).

**Figure 1:**
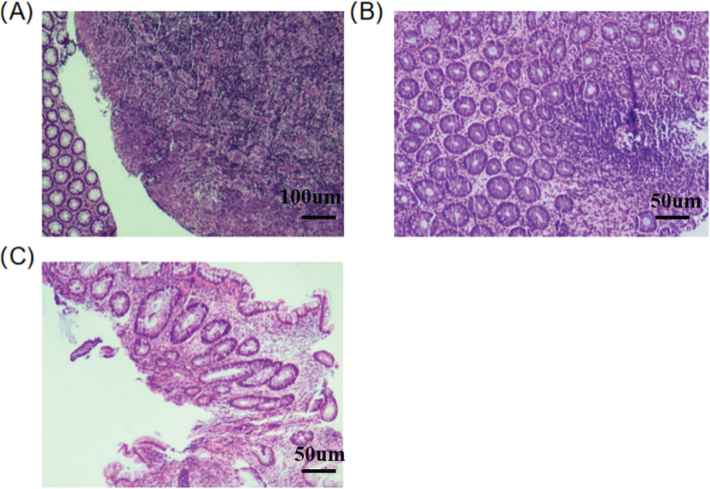
The pathological image of HGD in patients with UC (a), the pathological image of LGD in patients with UC (b), and the pathological image of IND in patients with UC (c).

Diagnostic criteria for dysplasia: The patient was diagnosed with dysplasia in the *Recommendations on Pathological Diagnosis of Inflammatory Bowel Disease Associated Dysplasia* formulated by the Gastroenterology Branch of the Chinese Medical Association (15): At least one biopsy should be taken from the lesion during colonoscopy for UC patients. If no obvious ulcers or erosions are observed by naked eye during colonoscopy, at least one biopsy should be taken from the rectum. The biopsy specimens should be diagnosed by professional pathologists. Through pathological examination, dysplasia can be classified into uncertain dysplasia (IND), low-grade dysplasia (LGD), and high-grade dysplasia (HGD) (Figure 1).

Diagnostic criteria for recurrence: The patient meets the diagnostic criteria for recurrence as stipulated in the *Chinese Clinical Practice Guideline on Management of Ulcerative Colitis (2023, Xi’an)* (14). After the patient enters the remission period either naturally or through drug treatment, the symptoms of UC recur. The most common symptom is bloody stool, and diarrhoea is also quite common. These can be confirmed through colonoscopy.

### Inclusion and exclusion criteria

Inclusion criteria: 1) The patient was diagnosed with UC (14); 2) Aged 18 years or older; 3) Complete clinical and follow-up data for the patient; and 4) Informed consent form signed by the patient and a family member.

Exclusion Criteria: 1) Pregnant or lactating women; 2) Patients with combined gastrointestinal perforation and toxic megacolon; 3) Patients with coagulation disorders; 4) Patients with immunodeficiency; and 5) Patients who cannot tolerate colonoscopy and computed tomography enterography (CTE) examination-related contraindications.

### Research methods

#### Collection of general data and clinical data

The patients’ general and clinical information was gathered through inquiries, medical records and laboratory reports, comprising gender, age, body mass index (BMI), disease course of UC, family history of colon cancer, drug treatment situation (mesalazine, azathioprine, infliximab, no medication), CTE imaging image features, disease classification, disease severity, lesion range, degree of disease activity, laboratory examination indicators, and grade of dysplasia.

1. CTE imaging features: A CTE examination was conducted on the patient within 24 h after admission. Before the CTE examination, patients are required to fast and orally take a negative contrast agent (such as mannitol) to fill the small intestine. During the examination, they lie on their backs and receive an intravenous injection of iodine contrast agent (inclusion of contraindications and signing of consent form is necessary). The contrast agent is injected using a high-pressure injector for multi-phase scanning, comprising arterial phase, portal venous phase, and delayed phase. Patients are instructed to hold their breath. The physicians collect images according to the thin-layer scanning and three-dimensional reconstruction protocols. Finally, the radiology doctor performs post-processing analyses such as multi-planar reformation. Record the imaging feature manifestations of the patient’s CTE, including pseudopolyps (visible polyp formation), bowel wall thickening (intestinal filling state shows intestinal wall thickness ≥ 4 mm), intestinal stenosis and intestinal shortening (intestinal tube dilation is insufficient when the intestinal tract is fully prepared), bowel wall stratification (visible widening of the intestinal mucosal sublayer, presenting as a ring-shaped low-density shadow), mesenteric vascular hyperplasia (compared with the adjacent intestinal tract, enhanced computed tomography [CT] shows obvious disorder and increase of intestinal wall vessels at the mesentery), mesenteric lymphadenopathy (thickness ≥ 5 mm), submucosal bubble (low-density small bubbles can be observed in the intestinal mucosal sublayer), mucosal abnormal enhancement (enhanced scan shows significant enhancement of the mucosal layer of the affected intestinal segment), mesenteric fat infiltration (increased density and blurring of the fat space around the intestinal tube), and loss of haustra (16, 17).
2. Disease classification: It is divided into the initial type, the chronic relapsing type, the chronic continuous type, and the acute fulminant type. 1) Initial type: It refers to the first occurrence without any previous history; 2) Chronic relapsing type: The active period alternates with the remission period; 3) Chronic continuous type: The initial attack lasts for a period of time with varying degrees of diarrhoea and bloody stool, lasting for more than 6 months, and may be accompanied by acute attacks; 4) Acute fulminant type: The onset is sudden, with bloody stool ≥ 10 times per day, accompanied by systemic toxic symptoms.
3. Disease severity: The disease activity degree is evaluated based on the modified Truelove and Witts disease severity and classification criteria. It is categorised as mild, moderate, or severe according on the patient’s haemoglobin (HGB), erythrocyte sedimentation rate (ESR), pulse, body temperature, frequency of bowel movements, and degree of haematochezia. Mild: The frequency of diarrhoea is ≤4 times per day, with mild or no haematochezia, no fever, rapid pulse or anaemia, and a sedimentation rate of erythrocyte <20 mm/h; Severe: The frequency of diarrhoea is ≥6 times per day, accompanied by obvious mucoid bloody stool, with body temperature >37.8°C, pulse >90 beats/min, HGB < 100 g/L, and ESR > 30 mm/h; Moderate lies between the two.
4. Lesion range: According to the Montreal classification method, the lesion range is classified according to the greatest proximal extent of inflammation observed on colonoscopy. 1) Rectal type (E1): Disease confined to the rectum without proximal extension to the sigmoid colon; 2) Left-sided colon type (E2): Inflammation extends proximally from the rectum to a point distal to the splenic flexure; 3) Extensive colon type (E3): Widespread abnormalities that affect the whole colon and even the splenic flexure.
5. Degree of disease activity: The severity of disease activity was evaluated using the modified Mayo score (18). A score of ≤2 points with no individual score >1 point indicated a clinical remission period. A mild activity phase was denoted by a score of 3 to 5, a moderate activity period by a score of 6 to 10, and a severe activity period by a score of 11 to 12.
6. Laboratory examination indicators: Mainly include C-reactive protein (CRP), white blood cell (WBC), ESR, HGB, serum albumin (ALB), platelet (PLT), C-reactive protein/albumin ratio (CAR), and faecal calprotectin (FC).

#### Clinical follow-up

UC patients will undergo regular outpatient follow-ups every 3 months after discharge to monitor their recurrence status. The follow-up period will end on December 31, 2024.

### Statistical analysis

Data analysis was conducted by Statistical Package for the Social Sciences (SPSS) 27.0 software. (IBM SPSS Statistics 27.0. IBM Corporation, Armonk, New York, USA. https://www.ibm.com/products/spss-statistics) The Shapiro-Wilk (SW) test was used to check the normality of the data. Quantitative data that followed a normal distribution were expressed as mean ± standard deviation (x ± s), and analysis of variance (ANOVA) was adopted for comparisons among the three groups, with independent sample t-tests for pairwise comparisons between groups. Non-normal data were expressed as *M* (*Q_25_*, *Q_75_*), and rank sum tests were employed for comparisons between groups. Count data were expressed as *n* (%) and compared using the *χ*^2^ test. When the sample size was less than 40 or the theoretical frequency was less than 5, Fisher’s exact probability method was employed for analysis. Univariate logistic regression analysis and ridge regression analysis methods were employed to screen the influencing factors of dysplasia in UC patients. Spearman rank correlation coefficient was used to analyse the correlation between the grade of dysplasia, related influencing factors, and recurrence types. Statistical significance was defined as a *P* value of less than 0.05.

## Results

### Comparison of general data between the two groups of patients

As presented in Table 1, the two groups were comparable in general data, including gender, age, BMI, disease course of UC, family history of colon cancer, and drug treatment (*P* > 0.05).

**Table 1:** Comparison of general data between the two groups of patients.

Indicators	Dysplasia group (*n* = 58)	Non-dysplasia group (*n* = 290)	*t*/*χ*^2^	*P*
**Gender, *n* (%)**			0.12	0.729
Man	27 (46.6)	142 (49.0)		
Woman	31 (53.4)	148 (51.0)		
Age (year), mean ± SD	45.8 ± 13.0	43.3 ± 10.8	1.53	0.128
BMI (kg/m^2^), mean ± SD	25.5 ± 3.6	24.9 ± 3.3	1.21	0.227
Disease course of UC (year)	2.9 ± 0.4	2.8 ± 0.2	1.16	0.246
Family history of colon cancer, *n* (%)	5 (8.6)	32 (11.0)	0.31	0.580
Drug treatment situation, *n* (%)			0.32	0.978
Mesalazine	26 (44.8)	133 (45.9)		
Azathioprine	16 (27.6)	83 (28.6)		
Infliximab	15 (25.9)	69 (23.8)		
No medication	1 (1.7)	5 (1.7)		

BMI: body mass index; SD: standard deviation; UC: ulcerative colitis.

### Comparison of clinical data between the two groups of patients

The clinical data between the dysplasia and non-dysplasia groups were compared, and the results are shown in Table 2. There was no statistically significant difference in the levels of CRP, WBC, ESR, HGB, ALB, PLT, and CAR between the two groups (*P* > 0.05). However, statistically significant differences were observed in disease classification, disease severity, lesion range, degree of disease activity, and FC (*P* < 0.05).

**Table 2:** Comparison of clinical data between the two groups of patients.

Indicators	Dysplasia group (*n* = 58)	Non-dysplasia group (*n* = 290)	*t*/*χ*^2^	*P*
Disease classification, *n* (%)			31.41	<0.001
Initial type,	12 (20.7)	138 (47.6)		
Chronic relapsing type	28 (48.3)	91 (31.4)		
Chronic continuous type	11 (19.0)	37 (12.8)		
Acute fulminant form	7 (12.0)	24 (8.2)		
Disease severity, *n* (%)			178.64	<0.001
Mild	3 (5.2)	231 (79.6)		
Moderate	10 (17.2)	39 (13.5)		
Severe	45 (77.6)	20 (6.9)		
Lesion range, *n* (%)			126.07	<0.001
E1	8 (13.8)	214 (73.8)		
E2	19 (32.8)	67 (23.1)		
E3	31 (53.4)	9 (3.1)		
Degree of disease activity, *n* (%)			156.82	<0.001
Clinical remission	5 (8.6)	211 (72.8)		
Mild active stage	10 (17.2)	61 (21.0)		
Moderately active stage	17 (29.4)	13 (4.5)		
Severely active stage	26 (44.8)	5 (1.7)		
Laboratory examination indicators, mean ± SD				
CRP (ug/mL)	11.6 ± 2.1	11.2 ± 1.9	1.30	0.194
WBC (10^9^/L)	8.5 ± 0.6	8.4 ± 0.6	1.02	0.308
ESR (mm/h)	17.1 ± 1.4	16.8 ± 1.3	1.62	0.106
HGB (g/L)	140.0 ± 11.3	137.8 ± 9.7	1.56	0.120
ALB (g/L)	43.6 ± 3.0	44.5 ± 3.7	−1.68	0.094
PLT (10^9^/L)	286.5 ± 30.3	279.6 ± 25.6	1.81	0.071
FC (ug/g)	377.9 ± 65.3	240.3 ± 68.1	14.15	<0.001
CAR	0.3 ± 0.1	0.1 ± 0.1	1.72	1.000

SD: standard deviation; CRP: C-reactive protein; WBC: white blood cell; ESR: erythrocyte sedimentation rate; HGB: haemoglobin; ALB: serum albumin; PLT: platelet; FC: faecal calprotectin; CAR: C-reactive protein/albumin ratio.

### Comparison of CTE imagological characteristics between the hyperplastic group and the non-hyperplastic group

**Figure 2:**
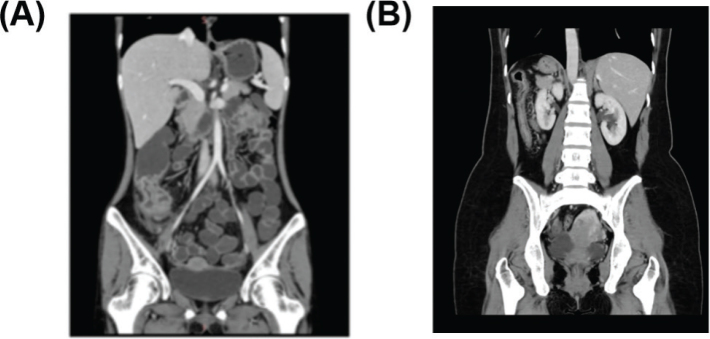
The CTE imaging image of dysplasia in patients with UC (a) and the CTE imaging image of non-dysplasia in patients with UC (b).

The typical imaging images observed in the two groups are provided in Figure 2. As could be seen from Figure 2a, patients with UC accompanied by dysplasia had typical imaging features such as pseudopolyps, intestinal stenosis, and bowel wall thickening. From Figure 2b, it could be observed that patients with UC without dysplasia had diffuse bowel wall thickening, mucosal enhancement, and absence of palatal folds, among others. The types and specific characteristics of CTE imagological examination in patients with dysplasia are shown in Table 3. Patients with dysplasia often presented with mesenteric lymphadenopathy, mucosal abnormal enhancement, and bowel wall thickening.

**Table 3:** Performance types and specific characteristics of computed tomography enterography imagological examination of patients in dysplasia group.

Performance type	Specific characteristic	Number of cases (*n* = 58)
Bowel wall changes, *n* (%)	Bowel wall thickening	51 (87.9)
	Bowel wall stratification,	47 (81.0)
	Mucosal abnormal enhancement	52 (89.7)
	Submucosal bubble	42 (72.4)
Colonic configuration alterations, *n* (%)	Loss of haustra	35 (60.3)
	Intestinal stenosis/Intestinal shortening	23 (39.7)
	Pseudopolyps	20 (34.5)
Extramural changes, *n* (%)	Mesenteric vascular hyperplasia	44 (75.9)
	Mesenteric fat stranding	25 (43.1)
	Mesenteric lymphadenopathy	52 (89.7)

### Univariate logistic regression analysis of dysplasia in UC patients

Variables showing significant differences in clinical data were incorporated into the logistic regression model (Quantitative assignment: Dysplasia: yes = 0, no = 1. Disease classification: initial type/chronic relapsing type = 0, chronic continuous type/acute fulminant type = 1. Disease severity: mild/moderate = 0, severe = 1. Lesion range: E1/E2 = 0, E3 = 1. Degree of disease activity: clinical remission/mild active stage = 0, moderately active stage/severely active stage = 1). The findings of the analysis are displayed in Table 4. Disease classification, disease severity, lesion range, degree of disease activity, and FC were independently correlated with the occurrence of dysplasia in UC patients using univariate logistic regression analysis (*P* < 0.05).

**Table 4:** Univariate logistic regression analysis of dysplasia in ulcerative colitis patients.

Indicator	B	SE	*Wald χ* ^2^	OR	95% CI	*P*
Disease classification	3.941	0.409	92.743	51.481	23.083–114.817	<0.001
Disease severity	3.844	0.391	96.693	46.731	21.718–100.551	<0.001
Lesion range	3.579	0.429	69.640	35.848	15.466–83.091	<0.001
Degree of disease activity	3.769	0.386	5.079	43.319	20.321–92.345	<0.001
FC	0.029	0.0094	61.710	1.029	1.022–1.037	<0.001

OR: odds ratio; CI: confidence interval; FC: faecal calprotectin; SE: standard error.

### Ridge regression analysis of dysplasia in UC patients

Since some variance inflation factor *(VIF)* values of the independent variable indicators were greater than 10, this indicated the presence of multicollinearity issues among the independent variables. Therefore, the ridge regression model was constructed with the disease classification, disease severity, lesion range, degree of disease activity, and FC as independent variables, and whether the patient developed dysplasia was used as the dependent variable. A K value of 0.20 was used in the model. The analysis results are shown in Table 5. The study found that the *R*^2^ of this regression model was 0.621, indicating that disease classification, disease severity, lesion range, degree of disease activity, and FC could explain 62.10% of the reasons for whether the patient developed dysplasia. The regression coefficient values of disease classification, disease severity, lesion range, degree of disease activity, and FC were 0.213, 0.167, 0.084, 0.001, and 0.002, respectively, and the *P* values of all five were less than 0.001, meaning that chronic continuous type/acute fulminant type disease classification, the disease severity was severe, the lesion range was E3, the degree of disease activity was moderately active stage/severely active stage, and high FC level could accelerate the possibility of hyperplasia in UC patients.

**Table 5:** Ridge regression analysis of dysplasia in ulcerative colitis patients.

Indicator	Non-standardised coefficient		Standardised coefficient	*t*	*P*	Collinearity diagnosis	
	*B*	SE	*Beta*			VIF	Tolerance
Constant	−0.319	0.035	–	−9.230	<0.001	–	–
Disease classification	0.213	0.032	0.203	6.596	<0.001	8.802	0.116
Disease severity	0.167	0.027	0.174	6.121	<0.001	13.561	0.074
Lesion range	0.084	0.038	0.072	2.188	0.003	3.843	0.260
Degree of disease activity	0.001	0.026	0.114	4.355	<0.001	18.178	0.055
FC	0.002	0.000	0.340	11.783	<0.001	1.163	0.860
*R* ^2^			0.621				
*F*			111.898				
*P*			<0.001				

VIF: variance inflation factor; FC: faecal calprotectin; SE: standard error.

### Comparison of recurrence situations between the two groups of patients

The recurrence situations of the dysplasia group and the non-dysplasia group are shown in Table 6. The study found that the number of recurrence cases in the dysplasia group was 53, including 16 cases (30.19%) of sporadic type, 25 cases (47.17%) of recurrent type, and 12 cases (22.64%) of chronic continuous type; while the number of recurrence cases in the non-dysplasia group was only 14, including six cases (42.86%) of sporadic type, five cases (35.71%) of recurrent type, and three cases (21.43%) of chronic continuous type. There were statistically significant variations between the two groups in terms of the number and types of recurrence (*P* < 0.05).

**Table 6:** Comparison of recurrence situations between the two groups of patients.

Indicator	Dysplasia group (*n* = 58)	Non-dysplasia group (*n* = 290)	*t*/*χ*^2^	*P*
Recurrence, *n* (%)			198.72	<0.001
Yes	53 (91.4)	14 (4.8)		
No	5 (8.6)	276 (95.2)		
Recurrence type, *n* (%)			45.91	<0.001
Sporadic type	16 (30.2)	6 (42.9)		
Recurrent type	25 (47.2)	5 (35.7)		
Chronic continuous type	12 (22.6)	3 (21.4)		

### Correlation analysis of dysplasia and frequency of recurrence in UC patients

**Figure 3:**
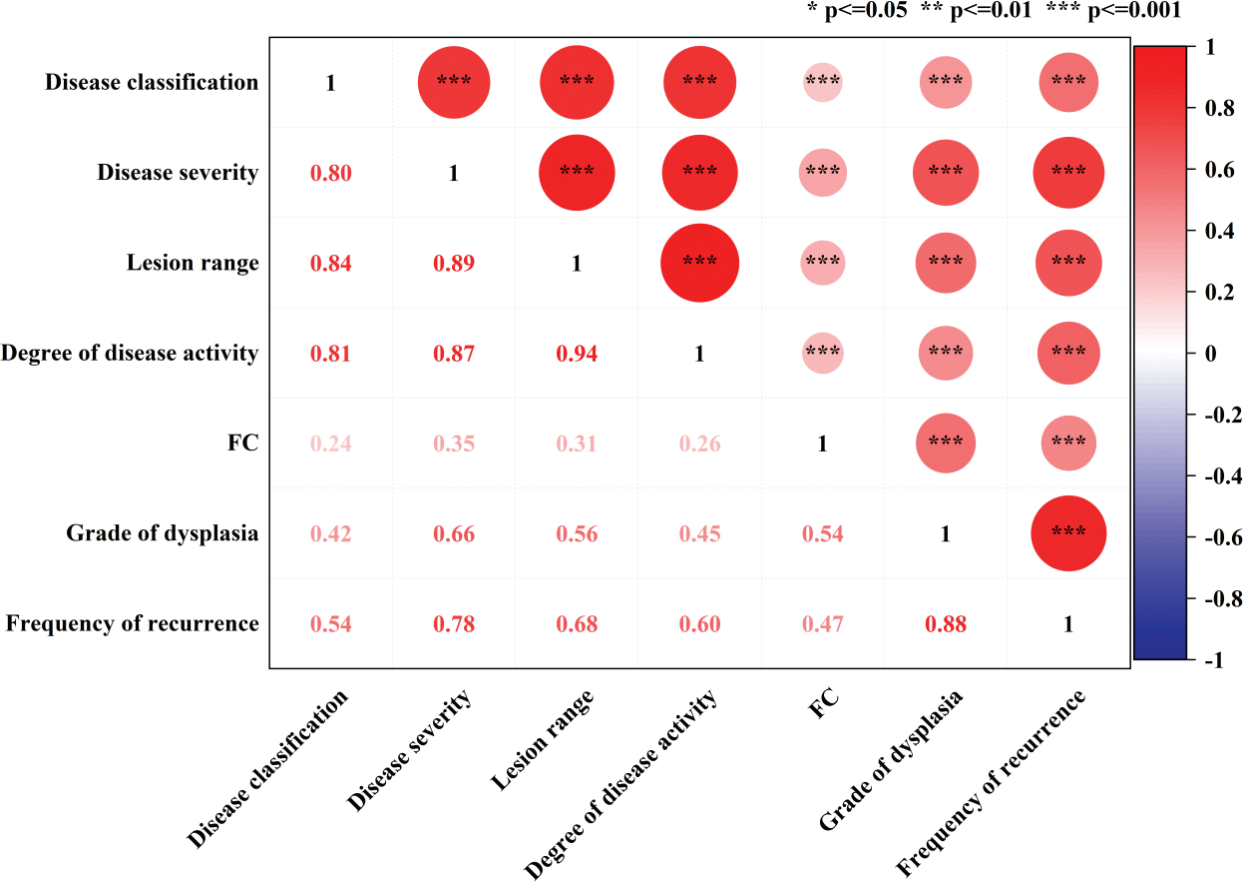
Correlation analysis heatmap of dysplasia (grade of dysplasia and influencing factors of dysplasia) and frequency of recurrence in UC patients.

The results of the correlation analysis between dysplasia (the grade of dysplasia and influencing factors of dysplasia) and the frequency of recurrence are shown in Figure 3. This correlation heatmap clearly revealed that the disease severity, lesion range, degree of disease activity, and grade of dysplasia were significantly positively correlated with the frequency of recurrence. Among them, the correlation with ‘grade of dysplasia’ was the strongest (with a correlation coefficient as high as 0.88). However, the association between the frequency of recurrence and FC was relatively weak.

## Discussion

UC is a chronic, non-specific IBD attributed to the interplay between genetic predisposition and environmental factors (19). The incidence of UC has been rising steadily in recent years, a trend that is closely associated with societal development and evolving lifestyles. In order to identify high-risk individuals as early as possible and reduce the impact of disease recurrence on UC patients. This study focused on exploring the influencing factors of dysplasia in UC patients, as well as the correlation between dysplasia and recurrence.

UC patients have a very high probability of developing dysplasia, leading to the abnormal cell proliferation becoming the focus of clinical attention (20). The risk of dysplasia in UC patients is influenced by various internal and external factors, and identifying these factors can help in precisely screening high-risk populations and optimising monitoring strategies (21). This study also found that disease classification, disease severity, lesion range, degree of disease activity, FC, and imaging features were independent influencing factors for dysplasia in UC patients through univariate logistic regression analysis. It is worth noting that previous studies have reported that the age of onset is an important variable influencing the disease progression (10). However, in this study, there was no age difference between the two groups of patients, indicating that in the study of newly diagnosed patients, the age of onset was not the main influencing factor for the occurrence of atypical hyperplasia in the short term. This might be related to the relatively short follow-up period of this study. The research also found that patients with dysplasia exhibited typical CTE imaging characteristics such as mesenteric lymphadenopathy, mucosal abnormal enhancement, and bowel wall thickening. The above research results are in agreement with previous findings by other researchers (22, 23), indicating that when patients develop dysplasia, the disease has entered the precancerous stage, and the risk of developing colorectal cancer for these patients significantly increases. Therefore, active intervention and management strategies need to be adopted. It was noteworthy that ridge regression analysis showed that disease classification, disease severity, lesion range, degree of disease activity, and FC level were all correlated with the development of dysplasia. Therefore, in clinical practice, more attention should be paid to these indicators. Patients with chronic continuous type or acute fulminant type were more likely to develop dysplasia, which was consistent with previous studies (6). UC patients with chronic continuous type and acute fulminant type, due to long-term or intense inflammatory stimulation, repeated damage and repair of intestinal mucosa, might activate signal pathways such as NOXs-ROS-p38MAPK, leading to abnormal epithelial cell differentiation and thereby increasing the risk of dysplasia (6). UC patients who had a more severe disease condition and a lesion grade of E3 had an increased likelihood of developing dysplasia. This result was consistent with previous studies (24, 25). This was mainly due to the fact that the extensive mucosa remains in a chronic inflammatory state for a long time, with a large affected intestinal area and intense and persistent inflammatory stimulation (26). This led to more frequent damage and repair processes of cells, making it easier for genetic material to undergo abnormal changes, thereby increasing the risk of dysplasia (27). In addition, UC patients whose disease activity was at the moderately active stage or severely active stage were also more likely to develop dysplasia. This was because the persistent inflammatory state activates multiple key signalling pathways (such as NF-κB, STAT3), which not only maintain the inflammation but also promote cell proliferation and inhibit normal apoptosis (28, 29). Furthermore, high FC levels were also independent risk factors for dysplasia. The research result was similar to the currently reported (30). The above phenomena indicated that FC could be used as an early warning indicator for dysplasia. FC, which is a sensitive marker for intestinal inflammation, had an elevated level that reflected the ongoing inflammatory activity. Persistent uncontrolled inflammation (with continuously elevated FC levels) can lead to DNA damage and abnormal cell proliferation, and may eventually progress to dysplasia (31). In summary, more attention should be paid to the disease classification, disease severity, lesion range, degree of disease activity, and the FC level of UC patients. Furthermore, the focus should be on identifying high-risk individuals (patients with chronic persistent type or acute fulminant type, patients with severe disease condition, patients with lesion grade of E3, patients with moderately active stage or severely active stage, and patients with high FC level) and reducing the probability of abnormal hyperplasia in patients.

UC results from a complex interplay of genetic susceptibility, immunity dysregulation, and environment triggers. The treatment emphasises individualised medication. For severe or complicated patients, surgical intervention is required, and at the same time, dietary, psychological and lifestyle management should be combined (32). This study revealed that patients in the dysplasia group exhibited a significantly higher recurrence rate than those in the non-dysplasia group, with sporadic and frequent types being the predominant patterns. Spearman correlation analysis further indicated that the frequency of recurrence was significantly associated with the grade of dysplasia and the influencing factors of dysplasia (disease severity, lesion range, degree of disease activity). The possible mechanisms for the above phenomenon were as follows: firstly, the persistent inflammatory microenvironment: the dysplasia area might secrete pro-inflammatory factors to maintain local inflammatory activity (33); secondly, destruction of the mucosal barrier function: dysplasia lead to abnormal expression of epithelial cell connection proteins, which aggravated intestinal permeability, promoted pathogen invasion and immune activation (34); thirdly, resistance to treatment: patients with HGD had a poorer response to traditional drugs (such as mesalazine), which might be related to the activation of tumour-related pathways (35). To sum up, dysplasia was significantly correlated with the recurrence rate and frequency of recurrence in UC patients.

In conclusion, disease classification, disease severity, lesion range, degree of disease activity, and FC level were the key influencing factors for the occurrence of dysplasia in UC patients, and dysplasia would increase the risk of disease recurrence. This study provides certain references for the monitoring and treatment of UC patients. For UC patients with lesion range E3 who present with a severe disease condition, including chronically active or acute fulminant disease activity, moderate to severe clinical activity, and high FC levels, enhanced monitoring and early intervention should be carried out to reduce the risk of dysplasia and recurrence.

This study is subject to several limitations: Firstly, the retrospective inclusion of sample size might introduce selection bias. Secondly, the limited sample size might lead to certain deviations in result analysis. Furthermore, when analysing the recurrence of patients, this study lacked longer-term continuous observation. Future studies should expand the sample size and prolong the observation period to guarantee the universality and dependability of the study findings.

## Conclusion

This study found that patients with dysplasia usually exhibited CTE imaging characteristics of mesenteric lymphadenopathy, mucosal abnormal enhancement, and bowel wall thickening. Chronic persistent type/acute fulminant type, severe disease condition, lesion grade of E3, moderately active stage/severely active stage, and high FC level increased the risk of dysplasia in UC patients. In addition, studies have found that when patients had developed dysplasia, it led to an increased recurrence rate in the later stage. The grade of dysplasia, disease severity, lesion range, and degree of disease activity were positively correlated with the frequency of recurrence of UC patients.

## ORCID

Rongli Liu https://orcid.org/0009-0009-4523-7443

Jing Luan https://orcid.org/0000-0002-1722-193X

Qianbo Dong https://orcid.org/0000-0003-4746-1819

Mingwei You https://orcid.org/0009-0001-3861-8266

Yuanming Kang https://orcid.org/0009-0009-1287-7030

Wenyan Wang https://orcid.org/0009-0004-0644-8030

## Data availability

The data that support the findings of this study are available from the corresponding author upon reasonable request.

## Funding

This research was supported by the Medical Scientific Research Project of the Health Commission of Hebei Province (grant number: 20240504).

## Author contributions

Rongli Liu was responsible for the conceptualisation, methodology, acquisition of funding, and the writing of the original manuscript. Jing Luan, Qianbo Dong, and Mingwei You conducted the formal analysis, data curation, review and editing. Yuanming Kang and Wenyan Wang conducted the investigation, review and editing. Hongli Liu carried out the conceptualisation, review and editing. Liqun Yan carried out validation, supervision, project management, review and editing.

## Disclosure statement

The authors declare that they have no conflicts of interest.

## Ethical statement

This study complies with the requirements of the World Medical Association’s ‘Helsinki Declaration’, and relevant laws and regulations in China. It has been approved by the Ethics Review Committee of The Second Hospital of Hebei Medical University [Approval Number: 2025-R645]. Informed consent was waived by the Ethics Review Committee.
